# Activation of SIRT-1 Pathway by Nanoceria Sheds Light on Its Ameliorative Effect on Doxorubicin-Induced Cognitive Impairment (Chemobrain): Restraining Its Neuroinflammation, Synaptic Dysplasticity and Apoptosis

**DOI:** 10.3390/ph15080918

**Published:** 2022-07-24

**Authors:** Medhat Taha, Sara T. Elazab, Alaa. M. Badawy, Abdullah A. Saati, Naeem F. Qusty, Abdullah G. Al-Kushi, Anas Sarhan, Amira Osman, Amira E. Farage

**Affiliations:** 1 Department of Anatomy and Embryology, Faculty of Medicine, Mansoura University, Mansoura 35516, Egypt; alaabadawy1984@gmail.com; 2 Department of Anatomy, Al-Qunfudah Medical College, Umm Al-Qura University, Al-Qunfudhah 28814, Saudi Arabia; 3 Department of Pharmacology, Faculty of Veterinary Medicine, Mansoura University, Mansoura 35516, Egypt; sarataha1@mans.edu.eg or; 4 Department of Community Medicine and Pilgrims Healthcare, Faculty of Medicine, Umm Al-Qura University, Makkah 24382, Saudi Arabia; aaasaati@uqu.edu.sa; 5 Medical Laboratories Department, Faculty of Applied Medical Sciences, Umm Al-Qura University, Makkah 24382, Saudi Arabia; nfqusty@uqu.edu.sa; 6 Department of Human Anatomy, Faculty of Medicine, Umm Al-Qura University, Makkah 24382, Saudi Arabia; agkushi@uqu.edu.sa; 7 Department of Internal Medicine, College of Medicine, Umm Al-Qura University, Makkah 24382, Saudi Arabia; aasarhan@uqu.edu.sa; 8 Department of Histology, Faculty of Medicine, Kafrelsheikh University, Kafr Elsheikh 33511, Egypt; mero.osman@med.kfs.edu.eg; 9 Department of Anatomy and Embryology, Faculty of Medicine, Kafrelsheikh University, Kafr Elsheikh 33511, Egypt; amira_sm201161@yahoo.com

**Keywords:** cerium oxide nanoparticles, doxorubicin, chemobrain, oxidative stress, synaptic plasticity

## Abstract

Chemo fog is one of the most serious health concerns encountered by cancer survivors receiving doxorubicin (DOX)-based chemotherapy. Oxidative stress, neuroinflammation, apoptosis and impairment of synaptic plasticity are regarded as the key factors implicated in DOX-induced cognitive impairment. This research aimed to assess the possible neuroprotective effect of cerium oxide nanoparticles (CeNPs) against DOX-induced neurotoxicity. Forty-eight rats were divided into four groups (12 rats/group): control group, CeNPs group (received oral CeNPs solution (35 mg/kg) daily for 4 weeks), and DOX group (were administered DOX intraperitoneally (2 mg/kg, once/week for 4 weeks)) and DOX+ CeNPs group. The findings revealed that CeNPs mitigated behavioral alterations in DOX-induced cognitive deficit. Additionally, CeNPs alleviated the histopathological abnormalities in hippocampus and ameliorated DOX-induced neuroinflammation by downregulating the expression of NF-κB, TNF-α, IL-1β and IL6. In addition, CeNPs antagonized the apoptosis through reducing the protein expression of cytochrome c and caspase 3. In addition, it stimulated the antioxidant defense, as indicated by upregulating the expression of the Nrf2, HO-1 and PGC-1α genes. CeNPs improved synaptic plasticity via acting on the BDNF. These actions were related through the modification of SIRT-1 expression. Based on the aforementioned results, CeNPs antagonized the doxorubicin-induced neurodegeneration by its antioxidant, anti-inflammatory and antiapoptotic effects, alongside its SIRT-1 mediated mechanisms.

## 1. Introduction

One of the most serious complications facing cancer patients is chemobrain, which halts their memory and executive function, during or post-chemotherapy treatment [1]. Cognitive impairment symptoms have been reported in 75% of patients during chemotherapy treatment and 35% of patients after chemotherapy administration [2]. Chemobrain is mainly due to the affection of the structure of the hippocampus and prefrontal cortex, causing some psychological symptoms such as memory impairment, difficulty in language and concentration and reduced processing speed [3]. Breast cancer patients are considered more vulnerable to developing cognitive impairment than other cancer type patients as a result of chemotherapy [4]. Cognitive impairment is frequently noted in breast cancer patients during and after treatment and it is probably caused by several factors including the cancer itself, endocrine remedy, stress and the hormonal alterations occurring during menopause [5]. One of the chemotherapeutic agents implicated in inducing cognitive impairment is doxorubicin. [6].

DOXorubicin (DOX), an antitumor drug, is a member of the anthracycline antibiotic group, which was approved by the Food and Drug Administration (FDA) [7]. It exerts its anticancer effect through several mechanisms including the inhibition of topoisomerase II, intercalation of DNA and overproduction of massive reactive oxygen species (ROS) [8]. Despite its major role in combating breast cancer, DOX has numerous harmful side effects on various body organs, including nephrotoxicity, hepatotoxicity, cardiotoxicity and neurotoxicity, which limit its utilization [9]. In contrast to the former hypothesis, indicating that DOX cannot penetrate the blood–brain barrier (BBB), Nakaagawa et al. [10] and Sardi et al. [11] reported the presence of DOX in brain tissues after intravenous (IV) injection. Furthermore, it has been announced that DOX can accumulate in the brain, resulting in remarkable toxicity via a secondary mechanism through increased peripheral proinflammatory cytokine TNF-α which crosses the BBB and hinders antioxidant defense leading to the death of neuronal cells, synaptic dysplasticity and eventually, chemobrain cognitive impairment [12,13]. However, further elucidation regarding the exact mechanism beyond DOX-induced cognitive impairment is needed.

Recent studies have declared that disruption in the metabolic process and mediators in the brain such as Sirtuins (SIRTs), Notch and glutamate play key roles in cognitive performance [14]. SIRT-1, an NAD+-dependent protein deacetylase, modulates multiple processes in the cell, including the metabolism [15], inflammation [16], aging [17] and apoptosis [18]. In addition, it has an important action in regulating memory and cognitive function through modulating the brain-derived neurotrophic factor (BDNF) which is involved in the differentiation of hippocampal neuronal cells and synaptic plasticity [19,20]. Moreover, SIRT-1 stimulates the production of antioxidant enzymes [21].

Cerium oxide nanoparticles (CeNPs) are among the most common metal oxide nanoparticles that are used in the synthesis of several industrial materials such as polishing glass material, optics manufacture and ultraviolet filters [22]. In addition, they are considered an important and lucrative material in biological disciplines including drug delivery, biomedicine, bioanalysis and bioscaffolding [23,24,25]. CeNPs have been used in several biological applications due to their powerful antioxidant character which depends on the ratio of Ce^3+/^Ce^4+^ on its surface. This antioxidant property mimics superoxide dismutase (SOD) and catalase (CAT) activities [23,26]. Nanoceria is regarded as one of the most valuable candidates for the treatment of diseases associated with oxidative stress such as neurodegeneration, diabetes and cardiovascular complications, owing to its prominent ROS-scavenging characteristics [27,28,29]. In addition, previous studies have recorded that nanoceria exerted a powerful anticancer effect through selectively causing oxidative stress in cancer cells and preparing them for radiotherapy without influencing the adjacent normal cells [30,31,32]. Moreover, CeNPs have been demonstrated to possess a strong anti-inflammatory activity [33,34]. Hegazy et al. [35] reported a neuroprotective effect of CeNPs against in 6-hydroxydopamine-induced Parkinsonism in rats. In addition, Saifi et al. [36] revealed that nanoceria has an ameliorating effect on cisplatin-induced nephrotoxicity in Swiss albino mice. Moreover, nanoceria has been proven to ameliorate the cardiotoxicity induced by DOX in mice [29].

Taking into account the available literature, we postulated that nanoceria, a potent antioxidant and anti-inflammatory compound, could be of beneficial value in antagonizing the neurotoxicity induced by DOX. This research was planned to investigate the potential neuroprotective effect of nanoceria on DOX-induced cognitive impairment and its role in the regulation of the SIRT-1 pathway.

## 2. Results

### 2.1. Effect of Nanoceria on Cognitive Function of the Hippocampus

**Figure 1:**
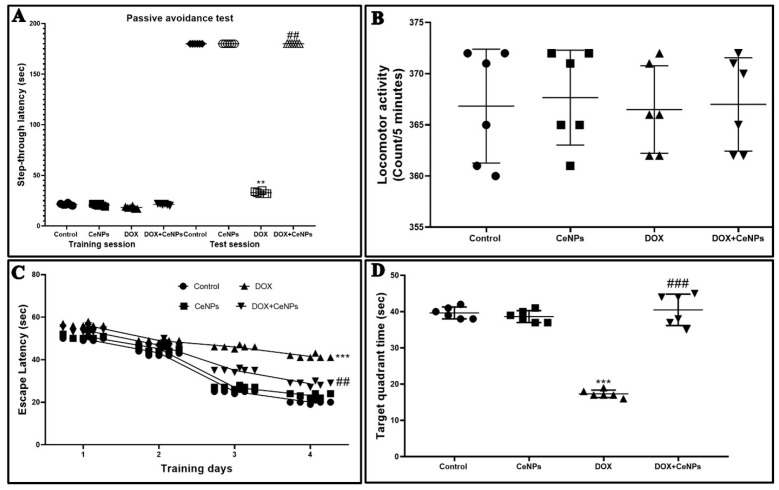
Effect of nanoceria treatment on DOX-induced behavioral changes: (**A**) Step-through passive avoidance for the training session and test session. (**B**) Locomotor activity. No significant difference was found between the different groups. (**C**) Morris water maze test (escape latency) during the four training days. Rats treated with DOX were slower in finding the platform. (**D**) Morris water maze test (time spent in target quadrant). DOX-treated rats spent less time in the target quadrant, while nanoceria co-treatment increased the time spent in the target quadrant. Passive avoidance data are elucidated as medians and interquartile ranges and analyzed using Kruskal–Wallis test followed by Dunn’s post hoc test. Meanwhile, results for (**C**) are presented as mean ± SD and statistically investigated utilizing two-way ANOVA followed by Bonferroni post-tests at *p* < 0.05. Data for (**B**,**D**) are showed as mean ± SD. Statistical comparison between the different groups was performed employing one-way ANOVA followed by Tukey’s post-test. *** *p* < 0.001 and ** *p* < 0.01 vs. control group, ^###^ *p* < 0.001 and ^##^ *p* < 0.01 vs. DOX group.

In the training session of the passive avoidance test, there was no significant difference between the experimental groups in the step-through latency. Meanwhile, in the test session, the step-through latency was significantly decreased (*p* < 0.01) in the DOX group in comparison with the control one. On the other hand, co-administration of CeNPs with DOX showed a significant increase (*p* < 0.01) in the step-through latency relative to rats administered DOX alone, indicating the improvement of the short-term memory by combined treatment with CeNPs (Figure 1A).

To exclude the impact of motor disability on the step-through latency of the passive avoidance test, we evaluated the influence of CeNPs and/or DOX on locomotor activity. The findings revealed no significant differences between different groups (Figure 1B).

We examined the spatial and learning memory of the rats by MWM test; the escape latency time showed no remarkable difference between different groups on the first and second days of the training. Meanwhile, on the third and fourth days, a significant increase (*p* < 0.001) in escape latency was observed in DOX-treated rats compared to the control rats. On the contrary, the groups which received CeNPs along with DOX showed a significant decrease (*p* < 0.001) in escape latency compared to the group administered DOX only (Figure 1C). Meanwhile, the time spent by the rats in the target quadrant in the probe test was significantly reduced (*p* < 0.001) in comparison to the control rats. In contrast, the co-treatment with CeNPs markedly increased (*p* < 0.001) the time spent in the target quadrant relative to the DOX group (Figure 1D).

### 2.2. Effect of Nanoceria on DOX-Induced Hippocampal Oxidative Stress and Gene Expression of Nrf2, HO-1 and PGC1-α

**Figure 2:**
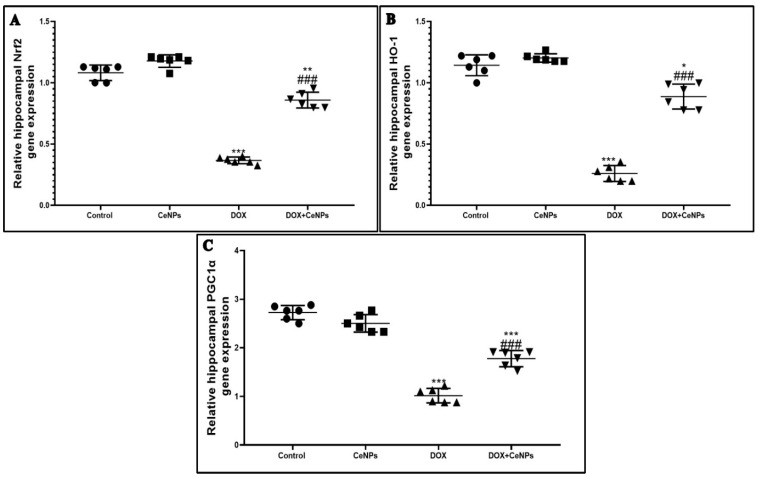
Effect of nanoceria treatment on relative gene expression of antioxidant genes. (**A**) Nrf2, (**B**) HO-1 and (**C**) PGC1-α in different groups of rats. All data were expressed as mean ± SD, (one-way ANOVA test followed by Tukey’s post hoc test, *** *p* < 0.001, ** *p* < 0.01 and * *p* < 0.05 vs. control group, ^###^ *p* < 0.001 vs. DOX group.

Administration of DOX induced a marked oxidative stress via decreasing the level of GSH andin addition to the activities of SOD and CAT and through producing significant elevation (*p* < 0.001) in the level of lipid peroxidation marker MDA, as compared to the control rats. Meanwhile, the administration of CeNPs in combination with DOX significantly elevated (*p* < 0.001) the level of antioxidant markers: GSH, SOD and CAT and showed a remarkable reduction in MDA level compared with the group which received DOX alone. The treatment with CeNPs showed a significant increase (*p* < 0.01) in the activities of SOD and CAT in relation to the control rats (Table 1). Moreover, the redox activity of CeNPs is explained by the significant elevation (*p* < 0.001) of the gene expression of the transcription factor Nrf2 and its antioxidant response elements HO-1 compared to the DOX group (Figure 2 A,B). Furthermore, the gene expression of PGC1-α, a marker of mitochondrial biogenesis, was significantly decreased (*p* < 0.001) in the DOX group in comparison with the control group. In contrast, concurrent administration of CeNPs with DOX significantly increased (*p* < 0.001) the gene expression of PGC1-α in relation to the DOX group (Figure 2C). CeNPs possess a powerful antioxidant property with a marked induction of endogenous antioxidant genes and improvement of the mitochondrial biogenesis.

### 2.3. Effect of Nanoceria on the Histoarchitecture of Hippocampal Tissue

**Figure 3:**
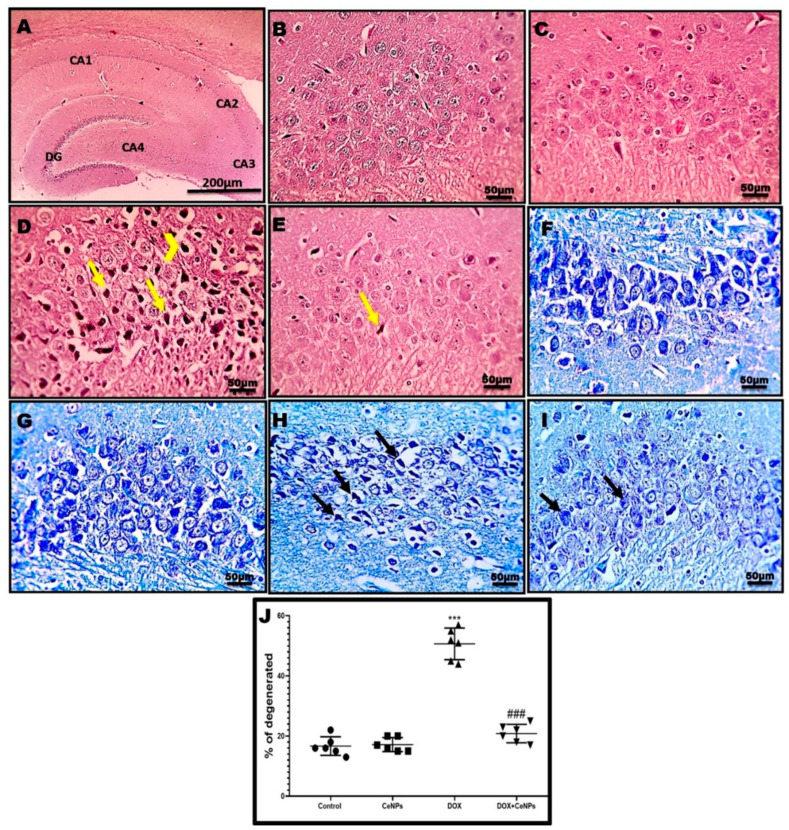
Effect of nanoceria on DOX-induced histopathological alterations. **Hippocampal sections stained with H&E**. (**A**) Hippocampus of control rat showing the dentate gyrus (DG) and the four regions of hippocampus CA1, CA2, CA3 and CA4 (Magnification ×40, Scale bar = 200 µm) (**B**,**C**) Normal neurons in pyramidal layer of CA3 region in both the control group and group that received CeNPs. (**D**) Hippocampal sections from the DOX group showed severe neuronal degeneration (yellow arrows) with prominent vacuolations (yellow arrowheads). (**E**) Hippocampal sections from treated group DOX + CeNPs showed very mild neuronal degeneration (yellow arrows). Magnification ×400, Scale bar = 50 µm. **Hippocampal section stained with toluidine blue stain**. (**F**,**G**) Hippocampal sections of control group and group which received CeNPs showing normal, blue-stained neurons in pyramidal layer (CA3). (**H**) Hippocampal sections from DOX group showed increased bluish staining due to many degenerated neurons (black arrows). (**I**) Hippocampal sections from treated group DOX + CeNPs showed a decreased bluish staining due to fewer degenerated neurons (black arrows). (**J**) Histogram of toluidine blue staining across six different fields from 3 rats per experimental group expressed as % of degenerated neurons compared to the total number of neurons. All data were expressed as mean ± SD. The analysis of the data was conducted employing one-way ANOVA test followed by Tukey’s post hoc test, *** *p* < 0.001 vs. control group, ^###^ *p* < 0.001 vs. DOX group. Magnification ×400, Scale bar = 50 µm.

**Figure 4:**
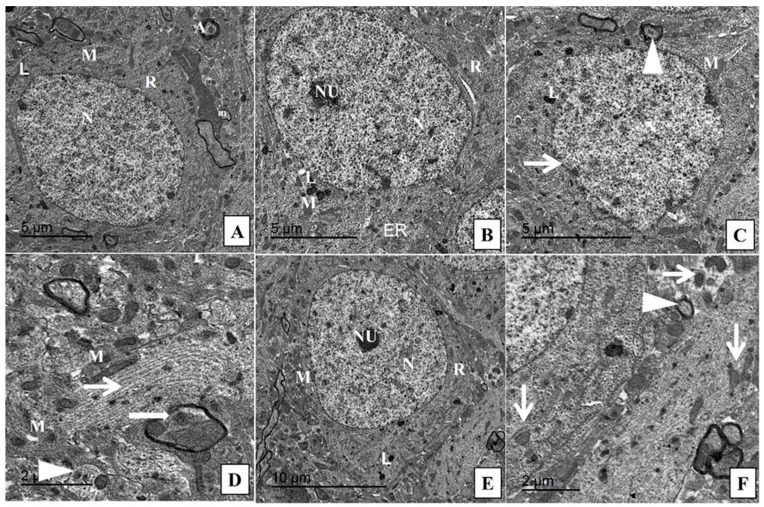
Transmission electron micrographs of hippocampus from different groups. (**A**) Control group showing the normal structure of the neuron with normal nucleus (N) and cytoplasm containing mitochondria (M), ribosome (R) with surrounding healthy myelinated axons wrapped by myelin (A) and axonal mitochondria (m). (**B**) CeNPs group showing the normal structure of the neuronal cells with intact nuclear envelope, distinct nucleoli, normal cytoplasmic organelles (mitochondria (M), ribosomes (R), lysosomes (L) and endoplasmic reticulum (ER)). (**C**) DOX group showing enlarged nucleus with blebs of nuclear envelope (thin arrow), loss of nucleolus and few irregular shaped mitochondria (M) with minimal degenerating sheaths surrounding a degenerated axon (arrowhead). (**D**) DOX group showing complete loss of myelin sheath (arrowhead), other area showing intact axon with gradual loss of myelin (thick arrow) beside dilated microtubules (thin arrow) intercalated with few irregular shaped mitochondria (M). (**E**,**F**) Treated group DOX + CeNPs showing the normal architecture of nucleus (N) with prominent nucleoli (NU) and most cytoplasmic organelles (mitochondria (M), lysosome (L) and ribosome (R) within the neurological cell. (**F**) Higher power showing with minimal mitochondrial membrane losses (thin arrows) either inside and around the neuronal cell with minimally affected sheath (arrowhead). Scale bar = 5 µm (**A**–**C**), 2 µm (**D**,**F**), 10 µm (**E**).

Histological examination of hippocampal tissue by a H&E stain revealed the normal structure of the neurons on the pyramidal cell layer of CA3 regions in the rats of the control and CeNPS groups (Figure 3B,C). In the DOX group, the neurons showed marked degeneration in the form of vesicular nuclei and these degenerated nuclei replaced by vacuolations (Figure 3D). To a great extent, the group which received CeNPs concurrently with DOX showed a marked decrease in degenerated neurons (Figure 3E). These findings were confirmed by the toluidine blue staining of CA3, which revealed an increase in the blue staining of degenerated neurons in the DOX group (Figure 3H), which was interestingly reversed in the DOX + CeNPs group (Figure 3I). These results are statically emphasized by increasing the percentage of the degenerated neurons in the DOX group (*p* < 0.001) in comparison with the control group and decreasing this percentage (*p* < 0.001) in the DOX + CeNPs group in relation to the DOX group (Figure 3J). Furthermore, the ultrastructural examination of the hippocampal tissue of the control and CeNPs groups showed a normal structure of the neuron, with a normal nucleus with distinct nucleoli, normal cytoplasm organelles and healthy myelinated axons wrapped by myelin (Figure 4A,B). Meanwhile, the DOX group exhibited an enlarged nucleus with blebs of the nuclear envelope, loss of nucleolus, few irregular-shaped mitochondria with completely degenerated axons, loss of myelin sheath and dilated microtubules (Figure 4C,D). On the contrary, co-treatment with CeNPs improved the ultrastructure of the hippocampal neurons, which were elucidated in the form of a normal architecture of the nucleus, with prominent nucleoli and cytoplasmic organelles with minimal mitochondrial membrane loss and lessaffected myelin sheaths (Figure 4E,F). The above findings indicated that treatment with CeNPs greatly improved the disturbed hippocampal histoarchitecture that resulted from DOX injection. These changes provid an explanation regarding the improvement in the cognitive behavior of the rats.

### 2.4. Effect of Nanoceria on DOX-Induced Hippocampal Neuroinflammation and Astrogliosis

**Figure 5:**
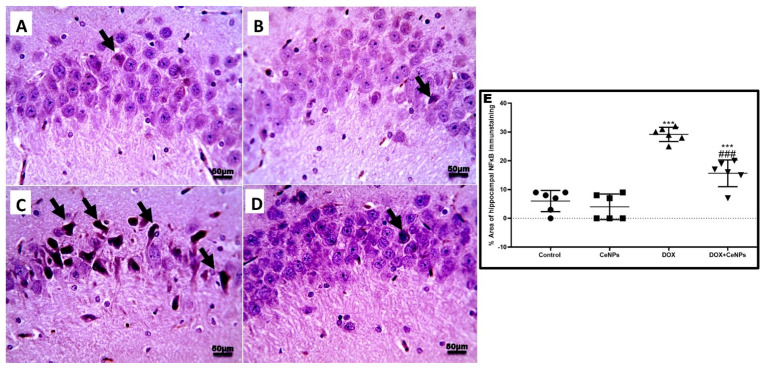
A. Microscopic pictures of immunostained sections of hippocampal sections against NFκB showing few numbers of positively brown stained neurons in pyramidal layer in (CA3) region in both (**A**,**B**) control group and the group that received CeNPs. (**C**) Hippocampal sections from DOX group showed markedly increased numbers of positively brown stained neurons (black arrows). (**D**) Hippocampal sections from treated group DOX + CeNPs showed decreased numbers of positively brown stained neurons (black arrows). (**E**) Histogram of % area of immunostaining hippocampal NFκB, values were expressed as mean ± SD. One-way ANOVA with Tukey post hoc analysis. *** *p* < 0.001 significant vs. control group ^###^ *p* < 0.001 significant vs. DOX group. Magnification ×400, Scale bar = 50 µm.

Immune expression of nuclear transcription factor NF-κB was significantly increased (*p* < 0.001) in DOX-treated rats in comparison with the control rats. Interestingly, the expression was significantly declined (*p* < 0.001) in combined treatment with CeNPs in comparison with the DOX group (Figure 5).

**Figure 6:**
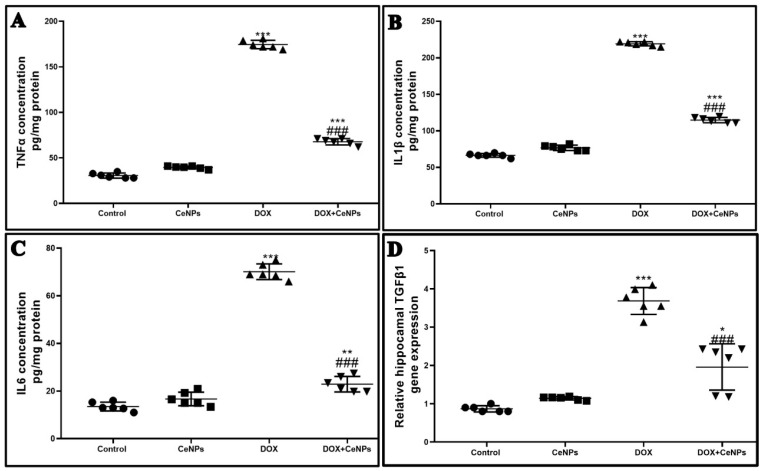
Effect of nanoceria treatment on proinflammatory markers measured by ELISA and on relative gene expression of TGF-β in different groups of rats. (**A**) TNFα protein expression. (**B**) IL1β protein expression. (**C**) IL6 protein expression. (**D**) relative gene expression of TGF-βin different groups of rats. All data are expressed as mean ± SD, (one-way ANOVA test followed by Tukey post hoc test was used for data analysis, *** *p* < 0.001, ** *p* < 0.01, and * *p* < 0.05 vs. control group, ^###^ *p* < 0.001 vs. DOX group.

**Figure 7:**
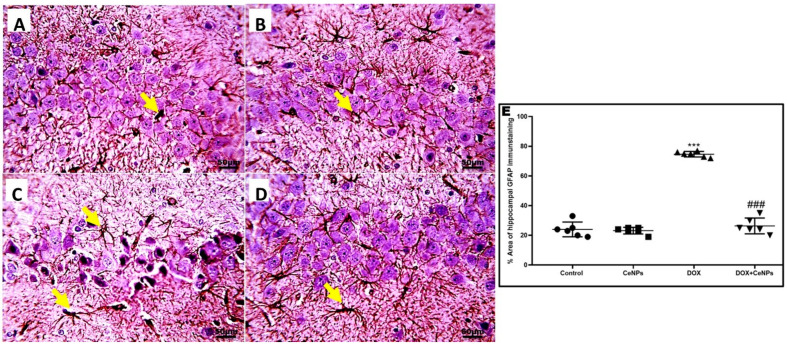
A. Microscopic pictures of immunostained sections of hippocampal sections against GFAP showing normal numbers of positively brown stained astrocytes in pyramidal layer (CA3) in the both (**A**,**B**) control group and group which received CeNPs. (**C**) Hippocampal sections from DOX group showed increased numbers and branches of positively brown stained astrocytes (yellow arrows). (**D**) Hippocampal sections from treated group DOX + CeNPs showed decreased numbers and branches of positively brown stained astrocytes (yellow arrows). (**E**) Histogram of % area of immunostaining hippocampal GFAP, values were expressed as mean ± SD. One-way ANOVA with Tukey’s post hoc analysis was performed. *** *p* < 0.001 significant vs. control group ^###^ *p* < 0.001 significant vs. DOX group. Magnification ×400. Scale bar = 50 µm.

Moreover, significant elevation (*p* < 0.001) of pro-inflammatory markers—TNFα, IL1β and IL6—and upregulation in the expression of the TGF-β gene was observed in the hippocampal tissue of the DOX-administered group, in contrast to the control rats (Figure 6A–D). In contrast, the groups receiving both DOX and CeNPs revealed a significant decrease (*p* < 0.001) in these pro-inflammatory markers and in the gene expression of TGF-β signaling compared to the group which was administered DOX alone. As a result of increased TGF-β gene expression, the immunoexpression of GFAP inflammatory astrocytes branched cells was significantly increased (*p* < 0.001) in the DOX group in comparison with the control group. Noticeably, the combined treatment with nanoceria significantly declined (*p* < 0.001) the expression of the inflammatory GFAP astrocytes compared to the DOX group (Figure 7). These results imply that CeNPs possess a strong anti-inflammatory property as it downregulates the gene expression of NFκB with a subsequent decrease in the production of the anti-inflammatory cytokines, as well as decreasing the gene expression of TGFβ with the downregulation of the reactive astrogliosis by decreasing the gene expression of the activated astrocytes. This anti-inflammatory property is strongly linked to its antioxidant activity.

### 2.5. Effect of CeNPs on DOX-Induced Hippocampal Neuronal Cells Apoptosis and Pyroptosis

**Figure 8:**
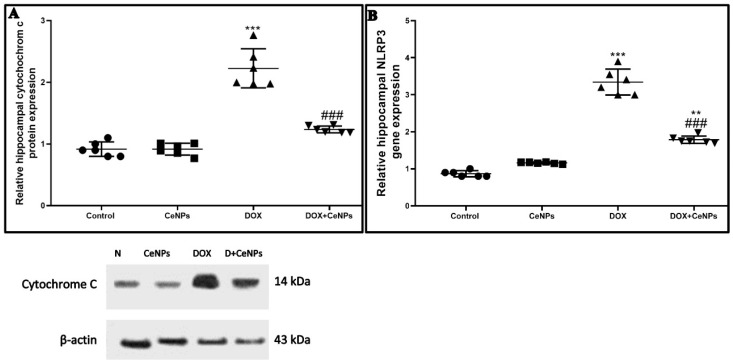
Effect of nanoceria treatment on the expression of cytochrome c protein and relative gene expression of NLRP3 in various groups of rats. (**A**) Cytochrome c protein expression as evaluated by Western bloating assay. (**B**) Relative gene expression of NLRP3. All data are elucidated as mean ± SD. One-way ANOVA test followed by Tukey’s post hoc test was used, *** *p* < 0.001 and ** *p* < 0.01 vs. control group, ^###^ *p* < 0.001 vs. DOX group.

**Figure 9:**
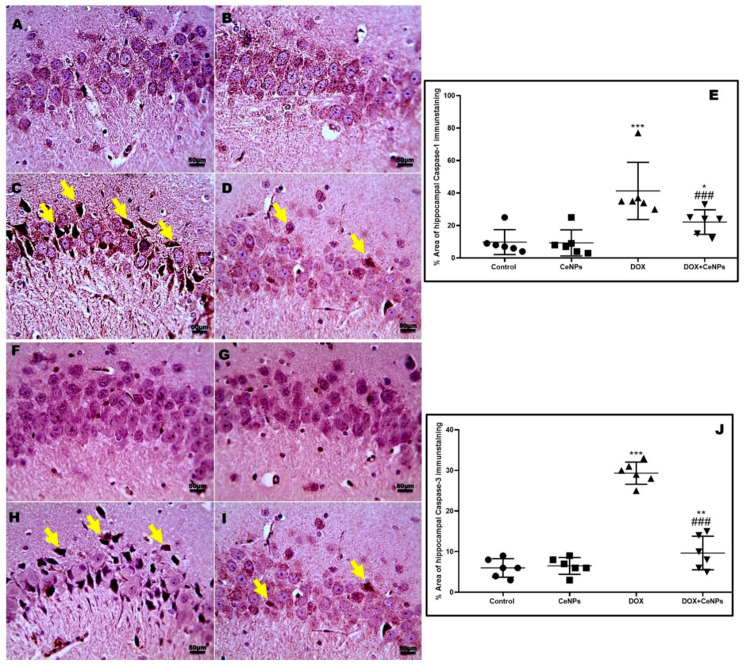
Effect of nanoceria on the hippocampal expression of caspase 1 and caspase 3 enzymes. (**A**,**B**,**F**,**G**) Hippocampal sections of control group and group which received CeNPs showing few numbers of positively brown stained neurons in pyramidal layer in CA3 region. (**C**,**H**) Hippocampal sections from DOX group showed markedly increased numbers of positively brown stained neurons (yellow arrows). (**D**,**I**) Hippocampal sections from treated group DOX + CeNPs showed decreased numbers of positively brown stained neurons (black arrows). (**E**,**J**). Histogram of % area of immunostaining hippocampal caspase 1 and caspase 3, respectively. Values are expressed as mean ± SD. The statistical investigation was carried out utilizing one-way ANOVA with Tukey’s post hoc analysis. *** *p* < 0.001, ** *p* < 0.01, and * *p* < 0.05 significant vs. control group ^###^ *p* < 0.001 significant vs. DOX group. Magnification ×400. Scale bar = 50 µm.

DOX treatment significantly raised (*p* < 0.001) the expression of cytochrome c protein, detected by Western blotting assay and the NLRP3 gene in relation to the control group. On the contrary, the co-treatment with CeNPs significantly decreased (*p* < 0.001) the protein expression of cytochrome c and downregulated the NLRP3 gene expression compared to the DOX group (Figure 8A,B). In addition, the immunoexpression of caspase 1 and caspase 3 enzymes were significantly increased (*p* < 0.001) in the DOX group in relation to the control one. Conversely, rats which received DOX plus CeNPs exhibited a marked decrease (*p* < 0.001) in their expression relative to the DOX group (Figure 9).

### 2.6. Effect of CeNPson Hippocampal Synaptic Plasticity

**Figure 10:**
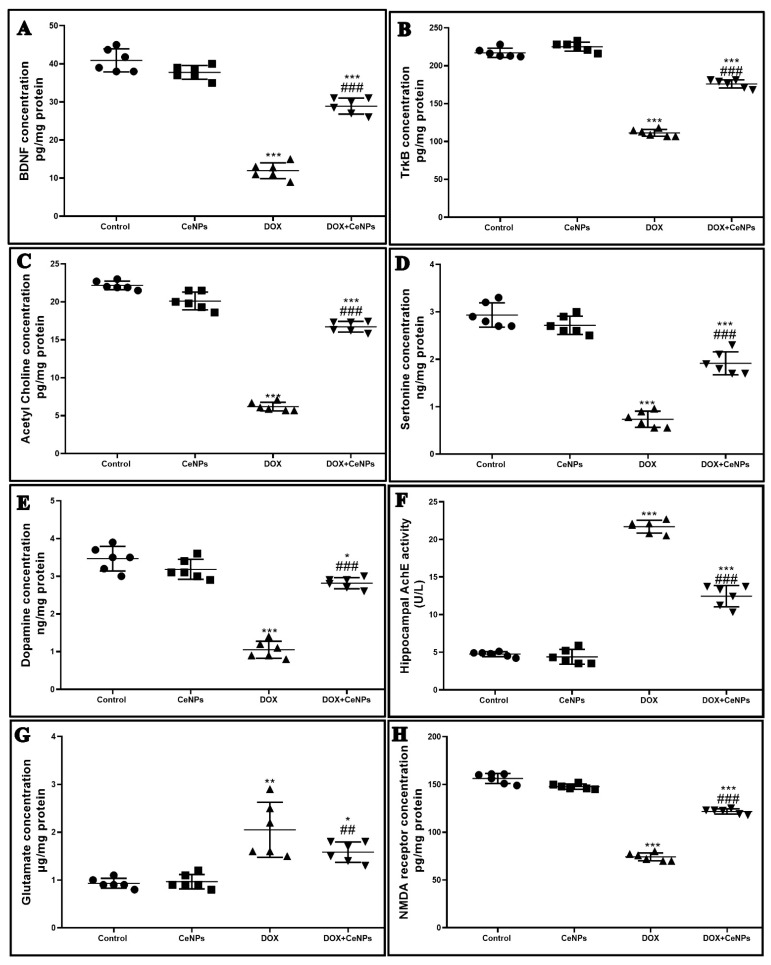
Effect of nanoceria treatment on protein expression of (**A**) BDNF, (**B**) TrkB, (**C**) acetylcholine, (**D**) serotonin, (**E**) dopamine, (**F**) AchE, (**G**), glutamate, and (**H**) NMDA receptor in different groups of rats. Data are presented as mean ± SD, *** *p* < 0.001, ** *p* < 0.01, and * *p* < 0.05 vs. control group, ^###^ *p* < 0.001 and ^##^ *p* < 0.01 vs. DOX group.

The protein expression of BDNF and its receptor TrkB concentrations were significantly decreased (*p* < 0.001) in the DOX group in comparison with the control rats. On the other hand, co-administration of CeNPs with DOX significantly increased (*p* < 0.001) their protein expression in relation to the DOX group (Figure 10A,B).

**Figure 11:**
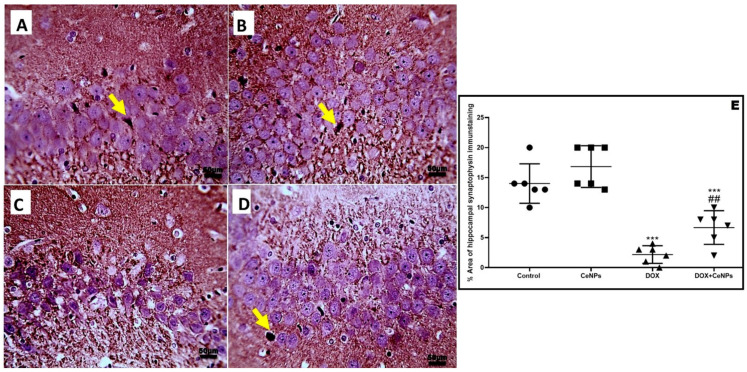
A. Microscopic pictures of immunostained sections of hippocampal sections against synaptophysin showing many positively brown stained neurons in pyramidal layer in CA3 region in both (**A**,**B**) control group and group which received CeNPs. (**C**) Hippocampal sections from the DOX group show negatively stained neurons. (**D**) Hippocampal sections from treated group DOX + CeNPs show increased numbers of positively brown stained neurons (yellow arrows). (**E**) Histogram of % area of immunostaining hippocampal synaptophysin. Values are shown as mean ± SD. One-way ANOVA with Tukey post hoc analysis was conducted. *** *p* < 0.001, ## *p* < 0.01. Magnification ×400. Scale bar = 50 µm.

The levels of the neurotransmitters—acetylcholine, serotonin, and dopamine in hippocampal homogenate—were significantly reduced (*p* < 0.001) in the DOX group relative to the control group. Meanwhile, combined treatment with CeNPs significantly increased (*p* < 0.001) their levels in comparison with the DOX group (Figure 10C–E). Additionally, compared to control group, the levels of acetylcholinesterase (AchE) and glutamate were remarkably (*p* < 0.001) increased in the DOX group. However, treatment with CeNPs along with DOX significantly downregulated (*p* < 0.001) the levels of AchE and glutamate compared to the DOX group (Figure 10F,G). The findings showed that the protein expression of the NMDA receptor in the hippocampal homogenate was significantly downregulated (*p* < 0.001) in the DOX group in relation to control group. Meanwhile, a significant upregulation (*p* < 0.001) in the protein expression of the NMDA receptor was observed in the DOX + CeNPs group compared to the DOX group (Figure 10H). In addition, the level of synaptophysin immunoexpression was significantly reduced (*p* < 0.001) in the DOX group relative to the control one, whereas co-treatment with CeNPs significantly upregulated (*p* < 0.01) the immunoexpression compared to the DOX group (Figure 11).

**Figure 12:**
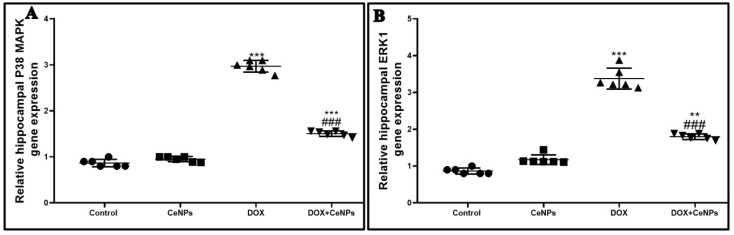
Impact of nanoceria administration on relative gene expression of P38 MAPK and ERK1 in different groups of rats. All data are presented as mean ± SD. The mean comparison was conducted employing by one-way ANOVA test followed by Tukey’s post hoc test, *** *p* < 0.001, ** *p* < 0.01, and * *p* < 0.05 vs. control group, ^###^ *p* < 0.001, ^##^ *p* < 0.01, and ^#^ *p* < 0.05 vs. DOX group.

The present study showed that the expression of the two memory-related kinase genes; P38 MAPK and ERK1 was significantly upregulated (*p* < 0.001) in the DOX group compared with the control one. Meanwhile, concurrent administration of nanoceria with DOX significantly downregulated (*p* < 0.001) the expression of the P38 MAPK and ERK1 genes in comparison with the DOX group (Figure 12A,B). Thus, the improvement in short-term memory as in the passive avoidance test and long-term memory as in the MWM test can now be clearly explained by the positive impact of CeNPs on synaptic plasticity in the form of the upregulation of the brain neurotrophic factor and the regulation of the level of neurotransmitters, as well as its regulatory effect on the two memory-related kinase genes.

### 2.7. Effect of CeNPs on Hippocampal SIRT-1

**Figure 13:**
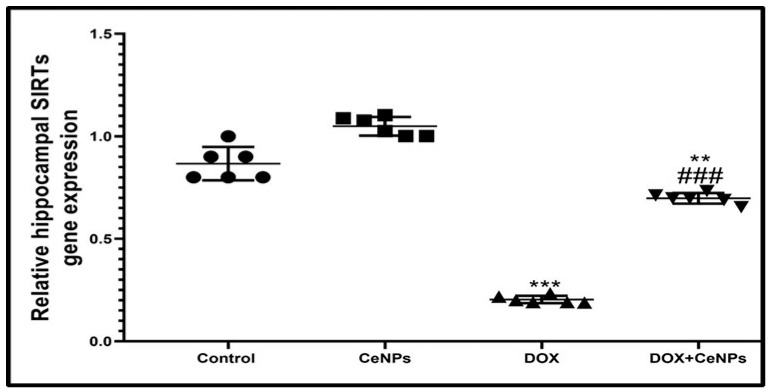
Effect of nanoceria treatment on relative gene expression of SIRT-1 in different groups of rats. Data are expressed as mean ± SD. One-way ANOVA test followed by Tukey’s post hoc test was used for data investigation, *** *p* < 0.001, ** *p* < 0.01, and * *p* < 0.05 vs. control group, ^###^ *p* < 0.001, ^##^ *p* < 0.01, and ^#^ *p* < 0.05 vs. DOX group.

The gene expression of SIRT-1 in hippocampal tissues was significantly decreased (*p* < 0.001) in the DOX group compared to the control rats. On the contrary, co-treatment with CeNPs significantly upregulated its expression (*p* < 0.001) compared to the DOX group (Figure 13).

## 3. Discussion

The present study revealed that systemic DOX administration every week for 4 consecutive weeks induced cognitive impairment as indicated by the increase in the time of stepdown latency of the rats in the passive avoidance test despite its normal locomotor activity, and increasing the escape latency time while decreasing the time spent by the rats in the target quadrant in the MWM test, suggesting that short-term memory and spatial learning are affected. These findings are consistent with those of Christie et al. [6], El-Agamy et al. [37], and Kitamura et al. [38]. As the hippocampus plays a central role in information consolidation from short- to long-term memory [39,40], these neurobehavioral changes are correlated with histoarchitecture alterations occurring in the hippocampus in the form of an increased percent of hippocampal neuronal degeneration and vacuolations. Conversely, co-treatment with CeNPs markedly improved the cognitive functions and short-term memory of rats, as evidenced by increasing the time of step-down latency and increasing the time spent by the rats in the target quadrant. This anti-amnestic effect of the CeNP may be owed to the amelioration of the histological changes in the hippocampus, as reflected by the decreasing percent of degenerated neurons. This neuroprotective effect of CeNPs is in line with the findings of Danish et al. [41], who stated that the intranasal administration of CeNPs markedly improved the cognitive function of the scopolamine-induced model of Alzheimer disease.

Despite the limited ability of DOX to cross the blood–brain barrier, its central neurotoxicity needs more elucidation [42]. Oxidative stress is the direct cornerstone in the pathogenesis of DOX-induced neurotoxicity. It has been reported that DOX exhibits severe oxidative stress [43], as the DOX undergoes a redox cycling process producing a large amount of superoxide reactive oxygen species disturbing the balance between the prooxidant and antioxidant in the cellular mitochondria, inducing neuronal degeneration and cognitive impairment [44]. In the same vein, the current study showed that DOX caused marked oxidative stress designated by reducing the activities of the antioxidant enzymes; SOD and CAT and the level of GSH and by increasing lipid peroxidation marker MDA, as well as downregulating the gene expression of the PGC1-α mitochondrial biogenesis regulator, Nrf2, a regulator of cellular resistance to oxidants, and its antioxidant response elements (HO-1). Our results are in accordance with those of Joshi et al. [45], who revealed that DOX-induced neurotoxicity was secondary to enhanced protein and lipid oxidation, leading to imbalance between the prooxidant and antioxidant with subsequent cellular injury. Moreover, Valle et al. [46] reported that DOX treatment induced oxidative stress by downregulating the expression of PGC1-α. In addition, Chen et al. [47] recorded that DOX possesses a downregulating effect on the Nrf2/Ho-1 pathway in cardiac tissues, causing serious oxidative insult. Based on the abovementioned findings, oxidative stress has a direct, key role in DOX-induced neurotoxicity. Interestingly, CeNPs administration along with DOX halts the oxidative stress by upregulating the antioxidant enzymes—GSH, SOD and CAT—and downregulating lipid peroxidation marker MDA, owing to their high antioxidant power, and their ability to cross blood–brain barrier [48,49]. The unique redox properties of CeNps qualifies it to be a promising treatment approach against DOX-induced chemobrain. The antioxidant character of CeNPs results from their ability to change from being oxidized to the reduced form (Ce3C and Ce4C) and vice versa [50]. The ratio between Ce^3 +^ and Ce^4 +^ on its surface activates its scavenging power to reactive oxygen species [27]. The nanoparticles’ size has a great influence on the proportion of the cerium ions Ce3+ and Ce4+ [51]. The fraction of redox active Ce3+ ions in the particles is inversely proportionate to the particle size. Hence, it was suggested to use a particle size of less than 30 nm to increase the percentage of Ce3+ valence status [23,52]. In addition, Zhou et al. [53] elucidated that CeNPs has catalase- and superoxide dismutase-like activities. Co-treatment with nanoceria upregulated the molecular level of PGC1-α, a strong regulator of mitochondrial biogenesis, Nrf2 and its antioxidant response element Ho-1, thus increasing the intrinsic antioxidant defense mechanism by increasing the production of the antioxidant enzymes. These findings are in agreement with those of Hasanvand et al. [54], who documented that treatment with nanoceria upregulated the Nrf2/Ho-1 pathway in the testes of diabetic rats.

DOX not only disrupts the antioxidant system but also triggers an inflammatory response as the peripheral circulating ROS stimulates redox responsive transcriptional factor NF-κB, which enhances the secretion of the proinflammatory cytokines TNFα, IL1β and IL6 [55,56]. From these cytokines, TNFα has the capability to cross the BBB [57], and further stimulates microglial astrocytes, which is the major constituent of the microglia and is involved in the regulation of cerebral microcirculation, synaptic transmission and the integrity of BBB [58]. Astrocyte activation enhances the release of more inflammatory cytokines which exaggerate neuroinflammation, producing a state of reactive gliosis, turning its function from beneficial to having a harmful effect which further intensifies the oxidative stress state and produces neuronal damage [59]. TGF-β is a pleiotropic cytokine that mediates the response of the cell to the inflammation [60]. Previous studies have discussed the role of TGF-β signaling in neuroinflammation, as it stimulates the proliferation and differentiation of the astrocytes [61,62]. In the present study, DOX injection markedly enhanced the immunoexpression of NF-κB, with subsequently increased proinflammatory cytokines TNFα, IL1β and IL6, and the immunoexpression of GFAP, an indicator of astrocyte activation, and the gene expression of TGF-β cytokine. Similar to the findings announced by Yan et al. [63], co-treatment with nanoceria produced a strong anti-inflammatory effect by downregulating the protein expression of NF-κB and its proinflammatory cytokines: TNFα, IL1β and IL6. In addition, nanoceria treatment downregulated astrocytes proliferation via decreasing TGF-β and astrogliosis marker GFAP expressions in line with the previous publication of [64]. NLRP3 is considered a cytosolic complex protein. Several studies have reported its impact on numerous neurological insults such as neuroinflammation [65]. NF-κB-mediated NLRP3 activations have been reported to trigger caspase-1, which stimulates IL-1β secretion [66]. In this research, DOX administration increased the gene expression of NLRP3 and protein expression of IL-1β. These findings are parallel with those of Wei et al. [67], who revealed the involvement of NLRP3 in the pathogenesis of DOX-induced cardiotoxicity. However, nanoceria co-treatment mitigated this deleterious effect by decreasing the hippocampal expression of NLRP3 and caspase-1, highlighting its anti-inflammatory effect. As far as the authors know, this study was the first to discuss the downregulating activity of nanoceria on the NLRP3 inflammasome pathway. This action may be due to the inhibitory effect of nanoceria on the NF-κB transcriptional factor.

DOX affected the hippocampal mitochondrial respiration, leading to mitochondrial impairment by the formation of mitochondrial permeability transition pores [68], resulting in mitochondrial swelling, with the rupture of its membrane and the subsequent release of proapoptotic protein cytochrome c, which stimulated apoptotic protein caspase 3 [69], eventually leading to the neuronal cells’ apoptosis and cognitive impairment. This experiment exhibited that DOX upregulated the protein expression of proapoptotic protein cytochrome c, with the overexpression of caspase 3 apoptotic neuronal cells, affecting synaptic plasticity and cognitive function. These results coincide with those of a previous study [70]. On the contrary, concurrent treatment with nanoceria downregulated the protein expression of cytochrome c and caspase 3, reversing the mitochondrial impairment and neuronal loss with a subsequent improvement in cognitive function and synaptic plasticity. These results are consistent with those of Solgi et al. [71], who revealed the antiapoptotic effect of nanoceria on the tissue of the testes of streptozotocin-induced diabetic rats.

Synaptic plasticity is affected by many of the brains’ growth factors, such as BDNF, which possess a key role in neurogenesis and neuronal cell differentiation [72]. In our study, the downregulated protein expression of BDNF and its receptor Trkβ reduced neurogenesis and neurodifferentiation, affecting synaptic plasticity. This finding is in accordance with the results of a previous report [73]. Co-administration of nanoceria reversed this effect of DOX. These results are in agreement with those of D’Angelo [74], who proved the neuroprotective effect of nanoceria against Alzheimer’s disease by improving neuronal cell survival through the activation of BDNF. In our study, DOX reduced the level of hippocampal acetylcholine and increased the level of acetylcholinesterase (AChE). These findings are in line with those published by Du et al. [75] and El-Agamy et al. [38]. It is well known that cholinergic neurons have a crucial role in hippocampal-mediated spatial learning and memory. Furthermore, its affection is involved in the pathogenesis of different dementias such as Alzheimer and Parkinson’s diseases [76]. DOX treatment activated the Ach enzyme which breaks down the acetylcholine into choline and acetic acid; this activation may be linked to DOX-induced oxidative stress [37]. The injection of DOX, in this study, downregulated two monoamine neurotransmitters, serotonin and dopamine, affecting the hippocampal-mediated long-term memory. These findings are similar to those of Kwatra et al. [77]. Serotonin plays an important role in hippocampal synaptic plasticity and its deficiency affects explicit memory, which is considered the hippocampal-mediated long-term memory of previously experienced items or events [78], and the dopamine neurotransmitter is involved in the long-term memory, especially during the encoding of the memory which demands hippocampal dopamine receptor stimulation [79]. In accordance with Thomas, [80], DOX injection elevated the level of hippocampal glutamate secretion from the astrocytes. This effect may be explained by the DOX-induced TNFα overproduction, which stimulates the astrocytes with the further overproduction of glutamate neurotransmitters [81]. Furthermore, excess glutamate stimulates extra synaptic NMDA receptors with the further inhibition of BDNF formation and its receptor Trkβ, affecting the synaptic plasticity, neurogenesis, neuronal differentiation and neuronal death [82]. Interestingly, treatment with CeNPs corrected the changes in the level of the neurotransmitters and receptors, as it elevated the level of hippocampal acetylcholine and decreased the activity of the acetylcholinesterase (AchE) enzyme in line with the report of Zhang et al. [83], who announced that polyacrylic acid-loaded CeNPs downregulated the activity of the AchE enzyme in the case of organophosphorus pesticides-induced neurotoxicity. This reduction in the enzyme activity in DOX + CeNPs group may be explained by its potent antioxidant property. At the same time, nanoceria increased the level of the two monoamines—serotonin (5HT) and dopamine—in a similar way to that found in the previous study of [36], the authors of which stated that treatment with 1 mg/kg of CeNPs increased the level of dopamine in 6-hydroxycobalamin-induced Parkinsonism, improving the dopaminergic neurons. We can explain this upregulating effect of the nanoceria on the two monoamines—serotonin and dopamine—by its high antioxidant power; this also may be due to the increase in the immunoexpression of synaptophysin, which is the presynaptic membrane protein responsible for carrying the neurotransmitters indicator for synaptic plasticity [84]. In contrast, CeNPs downregulated the glutamate neurotransmitters and its receptor NMDA. To the best of our knowledge, no previous studies discussed the impact of CeNPs on glutamate neurotransmitters and their receptor. This action may be attributed to the downregulating effect of nanoceria on TNFα, which led to the inhibition of astrocyte glutamate production. Correction of the level of the neurotransmitter by nanoceria with an improvement in the immunoexpression of synaptophysin shortened the time of passage of the nerve impulse, thus improving synaptic plasticity. Furthermore, this study demonstrated that DOX activated two memory-related kinase genes; P38MAPK and ERK1 which affect serotonin-mediated long-term facilitation, as P38MPAK mediates synaptic inhibition, while ERK1 mediates synaptic facilitation. However, the inhibitory effect of P38MAPK is superior to the faciliatory effect of ERK1 on the synaptic plasticity and the long-term facilitation of long-term memory [85]. In this research, DOX enhanced the gene expression of P38MAPK and ERK1. These results coincide with those of Liu et al. [86,87]. On the other hand, nanoceria co-treatment significantly downregulated the gene expression of the two kinases, in line with another study [88]. From all of the above findings, the neuroprotective effect of nanoceria against DOX was achieved through downregulating the expression of BDNF, disturbing the neurotransmitters and activating the two long-term memory-associated kinase.

Our study was concerned with examining the impact of SIRT-1 expression in the pathogenesis of DOX-induced neurotoxicity and the role of nanoceria in targeting it to provide a neuroprotective effect. SIRT-1 is considered a central regulator of metabolic function in the cells. In this research, the administration of DOX downregulated the gene expression of SIRT-1. In the same vein, Ruan et al. [89] mentioned that DOX downregulated SIRT-1 expression in cardiotoxicity. SIRTs-1 downregulation by DOX is explained by Guo et al. [90], who reported that TNFα activation downregulated SIRTs-1 expression. It was recorded that SIRT-1expression has several beneficial effects against DOX-induced neurotoxicity, as SIRT-1 enhanced expression quench oxidative stress by its activation of PGC1-α, a mitochondrial biogenesis marker, as well as scavenging ROS production, also upregulating Nrf2, a defense transcriptional gene that enhances the production of endogenous antioxidant enzymes [91]. In this context, SIRT-1 downregulated the expression of the NF-κB signaling pathway, thus inhibiting the secretion of the proinflammatory markers TNFα, IL-1β and IL6, in addition to NFκB involved in the expression of NLRP3 inflammasome and apoptotic caspase 3 gene expression stimulating apoptosis [92]. Moreover, SIRTs-1 is considered one of the regulators of synaptic plasticity and cognitive function by its regulation of BDNF expression [93] and inhibition of the glutamate and its NMDA receptor [94]. We are the first study, to the best of the authors’ knowledge, the abovementioned upregulating effect of nanoceria on SIRT-1.

## 4. Materials and Methods

### 4.1. Animals

This experiment was performed following the instructions of the US National Institutes of Health for laboratory animal care (NIH Publication No. 85-23, revised 2011), and was approved by the Medical Research Ethical Committee, Faculty of Veterinary Medicine, Mansoura University, Egypt (Code NO R/122). Forty-eight male Albino rats (180–230 g) were bought from AL Nile experimental research center, Mansoura, Egypt. Rats were housed in cages under suitable environmental conditions at 24 °C and were provided with standard food pellets and water ad libitum. Two weeks before the experiment, the rats were kept to become accustomed to the experimental environment.

### 4.2. Chemicals

Doxorubicin in the form of doxorubicin hydrochloride was procured from Hikma Pharmaceuticals, Giza, Egypt. Cerium oxide nanoparticles in powder form were supplied from Sigma-Aldrich Co. (St. Louis, MO, USA), with the CAS number 1306-38-3. Its particle size was < 25 nm. It was suspended in demineralized water at a concentration of 0.4 mg/mL [95].

### 4.3. Experimental Design

Rats were subdivided into four groups (12 rats/group): control group—rats were administered saline orally; CeNPs group—rats received 35 mg/kg of body weight of the CeNPs solution via a gastric tube daily for 4 consecutive weeks, a dose selected based on a previous work published by Khasksar et al. [96]; and the DOX group—rats were injected intraperitoneally (i.p.) with DOX (dissolved in saline) at a dose of 2 mg/kg/week at 0, 7, 14 and 21 days of the experiment, which lasted for 4 weeks. The dose of doxorubicin was adjusted according to El-Agamy et al. [37] and Kitamura et al. [38]; the DOX + CeNPs rats were administered 2 mg/kg/week of DOX i.p. plus 35 mg/kg/daily of CeNPs orally for 4 consecutive weeks.

### 4.4. Behavioral Tests

#### 4.4.1. Passive Avoidance Test

To test short-term memory, a passive avoidance test was carried out as previously described by Abdel-Aziz et al. [97]. The idea of the test was to examine the short-term memory of the rats in different groups; on the 28th day of the experiment, one week after the last dose of DOX, we started the training session, and the test session was performed 24 h later (on the 29th day of the experiment). The rats were gently placed in step-through passive avoidance apparatus (Ugo Basile, Italy); this apparatus has two compartments—one light and the other dark—with a sliding door in between, and during the test session, the rats stepped through dark compartment, putting their 4 paws on the gride floor. Just after the closure of the sliding door, electric shock with an amplitude of 1 mA was delivered for 2 s. The rats who failed to step through the dark compartment for 90 s were excluded from the test. On the next day, the day of the test session, we put the rats in the light room and the time of step latency to the dark room was recorded automatically, with no electric current as an indicator for the short memory of the rats after a noxious stimulus. The time of the test session was 3 min.

#### 4.4.2. Assessment of Locomotion

A meter (Opto-Varimex-Mini Model B; Columbus Instruments, Columbus, OH, USA) was used to assess the rats’ locomotor activity. The activity measurement depends on the interruption of the infrared beam frequency (wavelength = 875 nm, scan rate = 160 Hz, diameter = 0.32 cm, and spacing = 2.65 cm) by the movements of the rats. The locomotor activity during the test was calculated by counts/5 min, based on the method previously reported by El-Agamy et al. [37].

#### 4.4.3. Morris Water Maze (MWM) Test

The evaluation of the spatial learning and memory was performed following the method of Jang et al. [98]. We conducted the MWM test for 4 consecutive days for training and on the 5th day, for the test, we put the rats into the Morris water maze apparatus (302050-WM/1800, TSE Systems, Berlin, Germany), with a white circular pool (180 cm in diameter and 60 cm in height, filled with water at 26 ± 1 °C). The apparatus was divided into 4 equal quadrants with a hidden platform in one quadrant immersed 2 cm below the level of the water (not seen from the surface of the water). During the 4 days of training, the rats were allowed to go on 3 trials per day. The rats were placed in the wall of the pool facing each quadrant to swim in each of the different quadrants when the rats reached the quadrant of the hidden platform, stayed there for 30 s, and continued swimming; if the rats failed to reach the hidden platform after 90 s of swimming, we put it in the platform and left it to stay for 30 s. On the 5th day of the test, the probe trial test was performed; the platform was removed and the rats were put inside facing the quadrant of the platform and allowed to swim for 90 s in the pool, and the time latency spent by the rats in the quadrant which contained the hidden platform was recorded by a digital camera, indicating the spatial memory of the rats.

### 4.5. Samples Collection

After behavioral tests, which started on the 28th day of the experiments and ended on the 32nd day after the MWM test, the rats of different groups were euthanized by cervical dislocation; the whole brain was removed, and the hippocampus of the different groups was dissected in to 4 parts. The first part was prepared as a 5% (*w*/*v*) tissue homogenate in 0.9% saline solution. The homogenate was centrifuged and the supernatant was used for measuring the oxidative stress biomarkers. The second part was stored at −80 °C for molecular analysis. The third part was preserved in 10% formalin for histopathological and immunohistochemical investigations. The fourth part was fixed in 2.5% glutaraldehyde for electron microscopic examination.

### 4.6. Biochemical Analysis

*Assessment of hippocampal oxidative stress markers (SOD, GSH, MAD, CAT)*.

Biochemical examination of antioxidant enzymes reduced glutathione (GSH), SOD, CAT and lipid peroxidation markers (malondialdehyde (MDA)) were assessed using commercial kits purchased from Biodiagnostics Co., (Cairo, Egypt) following the guidelines of the manufacturers.

### 4.7. Histopathological Examination

After the excision of brain tissue, it was washed by PBS, fixed for 24 h in 10% formalin with PH 7.2, dehydrated by different grades of alcohol, immersed in a paraffin block at 56 °C in hot air for one day, and then cut by sledge microtome into a Section 4 μm in thickness, the hippocampal tissue was transferred to glass slides for hematoxylin and eosin (H&E) staining, and other slides were stained for 2 min with 0.1% toluidine blue and, after that, mounted with Canada balsam [99]. Then, the slides were examined using a light microscope. Regarding the toluidine blue staining, the hippocampal neurons were considered viable when their nuclei are rounded and their nucleoli are visible. On the contrary, when the cells were deeply stained and shrunken, it was considered non-viable. The degenerated neurons were counted in six nonoverlapped fields from three rats per experimental group. This was calculated as a percentage of the degenerated neuron in the high-power field in comparison to the total number of neurons in the hippocampus [38].

### 4.8. Immunohistochemical Assay of GFAP, NFκB, Caspase 3, Caspase 1 and Synaptophysin

Immunohistochemical examination was performed in accordance with [100]. The 4 μm-thick section of hippocampal tissues was deparaffinized by xylene then hydrated by different grades of alcohol, washed with distilled water. To block the endogenous peroxidase, the sections were incubated with 3% of H_2_O_2_ for 30 min; after that, it was washed by PBS and incubated with a primary antibody of glial fibrillary acid protein (GFAP) (anti-GFAP rabbit pAb, GB11096, Servicebio), nuclear factor- kB/p 65 (NF-κB p65) (NF-kB p65/RelA rabbit mAb, A19653, abclonal)caspase 3 (anti-active caspase 3 rabbit pAb, GB11532, servicebio) caspase 1 (caspase-1 Rabbit pAb, A0964, abclonal) and synaptophysin (synaptophysin rabbit pAb, A6344, abclonal), overnight at 37°C. Then, it was washed by PBS and incubated for 60 min with a secondary antibody (biotin-conjugated goat anti-rabbit IgG antiserum), then washed with PBS, and then incubated with conjugated streptavidin peroxidase for 30 min, antibody biotin-avidin peroxidase complex reacted with DAPA for 10 min, then was washed with PBS counterstained with H&E. A digital camera (Leica EC3, Leica, 148 Germany) connected to a microscope (Leica DM500, Leica, Germany) was used for picking up photomicrographs of the sections. The intensities of immunostaining were quantified using the Image J software (National Institutes of Health, Bethesda, 150 MD, USA). Six nonoverlapped fields in a high power field in 3 rats for each experimental group were used for the quantification of the immune expression percent area of GFAP, NFκB, caspase3, caspase1 and synaptophysin in the hippocampal CA3 region.

### 4.9. Transmission Electron Microscopy

Hippocampal tissue was fixed in 2.5% glutaraldehyde for 24 h; after that, it was postfixed with 1% osmium tetroxide for 2 h, then hydrated with different grades of alcohol embedded in epoxy resin; then, 1 μm semithin section was stained with toluidine blue stain and was examined with a light microscope to determine the target area for the examination. An ultrathin section of 70 nm was made by the use of a diamond knife on an LKB microtome on grades of copper. This was double stained with lead and uranyl acetate. The sections were examined by a JEOL-JEM-100 SX electron microscope at 80 kilo vol (Jeol Ltd., Tokyo, Japan) at the electron microscope unit in the Faculty of Agriculture, Mansoura University, Mansoura, Egypt [101].

### 4.10. RT-PCR Assessment

The hippocampal total RNA was extracted by Direct-zol RNA Miniprep Plus (Cat# R2072, ZYMO RESEARCH CORP, Irvine, CA, USA), according to the guidelines of the manufacturer; after that, it was quantified by a Beckman dual spectrophotometer (Pasadena, CA, USA). Reverse transcription of the extracted RNA was performed by SuperScript IV One-Step RT-PCR kit (Cat# 12594100, Thermo Fisher Scientific, Waltham, MA, USA). QRT-PCR was carried out using PCR. The 96-well plate StepOne instrument (Applied Biosystem, Waltham, MA, USA), PCR primer pairs used for nuclear erythroid-related factor-2 (Nrf2), heme oxygenase-1 (HO-1), peroxisome proliferator-activated receptor-gamma coactivator (PGC1-α), transforming growth factor beta (TGF-β), sirtuin (SIRT-1), P38 MAPK, ERK,1 and NLRP3, as well as the reference control glyceraldehyde-3-phosphate dehydrogenase (GAPDH) gene, are shown in (Table 2).

### 4.11. Western Blotting for Cytochrome c

The tissue of the hippocampus was homogenated on ice, and after that, lysed in a lysis buffer that contained 50 mM Tris-HCl (pH 7.5), 150 mM NaCl, 0.5% deoxycholic acid, 1% Nonidet P40, 0.1% SDS, 1 mM PMSF and 100 mg/mL leupeptin. The contents of the protein were assessed by Bradford Protein Assay Kit (SK3041) (Bio basic Inc, Markham, ON L3R 8T4, Canada). A total of 20 μg of the protein was separated on SDS-polyacrylamide gels which were transferred to a nitrocellulose membrane. After that, it was incubated with cytochrome c (Cyto-C, #4272, Cell Signaling Technology Inc., Danvers, MA, USA) and β-actin as a control protein (Cat. no. SC-69879, Santa Cruz Biotechnology, Dallas, TX, USA). Horseradish peroxidase-conjugated secondary antibodies were added. Thereafter, the blot was washed with TBST 3 times. The chemiluminescent substrate (Clarity TM Western ECL substrate Bio-Rad cat#170-5060) was applied to the blot. The signals of chemiluminescent were captured using a CCD camera-based imager. Image analysis software was used to read the band intensity of the target proteins against the control beta-actin by protein normalization on the ChemiDoc MP imager [102].

### 4.12. Enzyme-Linked Immunoassay (ELISA) for BDNF, Trkβ Receptor, Proinflammatory Cytokines (TNFα, IL-1β and IL6), Neurotransmitters (Acetylcholine, Acetylcholinesterase, Serotonin, Dopamine, GABA and Glutamate) and NMDA Receptor

After scarification, the tissue of the hippocampus was washed with ice and blotted between folds of filter paper. A total of 10% of hippocampal homogenate was prepared in 0.05 M phosphate buffer (pH 7) by a polytron homogenizer at 40 °C. Then, the homogenate was centrifuged for 30 min at 10,000 rpm and the supernatant was used to estimate BDNF, tropomysin-related kinase B (Trkβ receptor), tumor necrosis factor-alpha (TNFα), interleukin 1 beta (IL1β), interleukin 6 (IL6), acetylcholine, serotonin, dopamine, acetylcholinesterase enzyme, glutamate and N-methyl-D-aspartate (NMDA) receptor by commercial ELISA kits (SEA011Ra, NBP2-76777, abx050220, E-EL-R0012, SEA079Ra, E4454-100, E4454-100, DOU39-K01, E0724Ra, KA1909 and ER0669), respectively, according to the instructions of the company. The method of determination of the tissue supernatant protein by ELISA kits was previously described by [103]. Color absorbance was read at optical density (OD) at a range 490 to 630 nm using an Enzyme-Linked Immuno-Sorbent Assay (ELISA) plate reader (Stat Fax 2200, Awareness Technologies, Palm City, FL, USA).

### 4.13. Statistical Analysis

Normality of the data was checked by applying the Shapiro–Wilk test. The data were analyzed using Graph pad prism software, 8.0 (La Jolla, CA, USA). We used the Kruskal–Wallis test followed by Dunn’s post hoc test for analysis of the data of the passive avoidance test. On the other hand, the normally distributed data were analyzed by one-way ANOVA followed by the Tukey post hoc test and expressed as mean ± SD. additionally, the training session of the MWM test was analyzed by two-way ANOVA followed by the Bonferroni post hoc test, and *p* < 0.05 was considered statically significant.

## 5. Conclusions

Treatment with DOX as a chemotherapeutic drug has several harmful side effects; one of them is the affection of cognitive function. It induces oxidative stress, neuroinflammation and apoptosis of neuronal cells, as well as affecting the synaptic plasticity. On the contrary, treatment with nanoceria exerted a neuroprotective effect against DOX-induced neurotoxicity. It improved the cognitive function of the rats and the histological architecture of the hippocampus, alleviated the oxidative stress, mitigated neuronal cell apoptosis and improved synaptic plasticity by enhancing the brain-derived neurotrophic factor and regulating the neurotransmitters.

## Figures and Tables

**Table 1:** Effect of nanoceria on oxidative stress markers (GSH, SOD, MDA and CAT) in rats’ hippocampus.

	Control Group	CeNPs Group	DOX Group	DOX + CeNPs Group
**GSH (μmol/g tissue)**	1.49 ± 0.07	1.67 ± 0.03	0.51 ± 0.04 ***	1.29 ± 0.34 ^###^
**SOD (U/g tissue)**	158 ± 9.20	183 ± 6.30 **	75 ± 5.50 ***	124 ± 5.40 ***^, ###^
**MDA (nmol/g tissue)**	7.4 ± 0.51	6.7 ± 0.42	21.4 ± 0.58 ***	13.4 ± 0.46 ***^, ###^
**CAT (U/g tissue)**	2.7 ± 0.27	3.3 ± 0.20 **	1.2 ± 0.15 ***	1.9 ± 0.16 ***^, ##^

All the above table data are expressed as mean ± SD (*n* = 6 rats). One-way ANOVA with Tukey’s post hoc test was used for analysis of these data. *** *p* < 0.001 and ** *p* < 0.01 significant vs. control group, ^###^ *p* < 0.001 and ^##^ *p* < 0.01 significant vs. DOX group.

**Table 2:** Primer’s sequence of all studied genes.

	Forward Sequence	Reverse Sequence	Gene Accession Number
NLRP3	GTGGAGATCCTAGGTTTCTCTG	CAGGATCTCATTCTCTTGGATC	NM_001191642.1
P38 MAPK	CACAGCACCTCAGCAATGAT	AGGCCTATCTTCCCAGGAAA	NM_053842.2
ERK1	TCAAGCCTTCCAACCTC	GCAGCCCACAGACCAAA	XM_046421134.1
Nrf2	AGGACATGGAGCAAGTTTGG	TTGCCCTAAGCTCATCTCGT	NM_031789.2
HO-1	TCAGGTGTCCAGAGAAGGCTTT	CTCTTCCAGGGCCGTGTAGA	NM_012580.2
SIRT-1	GACGACGAGGGCGAGGAG	ACAGGAGGTTGTCTCGGTAGC	XM_006223877.1
TGF-β1	GACTCTCCACCTGCAAGACC	GGACTGGCGAGCCTTAGTTT	NM_021578.2
PGC1-α	ATCCTCTTCAAGATCCTGTTACT	CGTGCTCATTGGCTTCATAG	XM_032916070.1
GAPDH	CCTTCTCCATGGTGGTGAAGA	CACCATCTTCCAGGAGCGAG	NM_001394060.2

## Data Availability

Data is contained within the article.
