# Correction: Taha et al. Activation of SIRT-1 Pathway by Nanoceria Sheds Light on Its Ameliorative Effect on Doxorubicin-Induced Cognitive Impairment (Chemobrain): Restraining Its Neuroinflammation, Synaptic Dysplasticity and Apoptosis. *Pharmaceuticals* 2022, *15*, 918

**DOI:** 10.3390/ph17111533

**Published:** 2024-11-15

**Authors:** Medhat Taha, Sara T. Elazab, Alaa. M. Badawy, Abdullah A. Saati, Naeem F. Qusty, Abdullah G. Al-Kushi, Anas Sarhan, Amira Osman, Amira E. Farage

**Affiliations:** 1 Department of Anatomy and Embryology, Faculty of Medicine, Mansoura University, Mansoura 35516, Egypt; alaabadawy1984@gmail.com; 2 Department of Anatomy, Al-Qunfudah Medical College, Umm Al-Qura University, Al-Qunfudhah 28814, Saudi Arabia; 3 Department of Pharmacology, Faculty of Veterinary Medicine, Mansoura University, Mansoura 35516, Egypt; sarataha1@mans.edu.eg; 4 Department of Community Medicine and Pilgrims Healthcare, Faculty of Medicine, Umm Al-Qura University, Makkah 24382, Saudi Arabia; aaasaati@uqu.edu.sa; 5 Medical Laboratories Department, Faculty of Applied Medical Sciences, Umm Al-Qura University, Makkah 24382, Saudi Arabia; nfqusty@uqu.edu.sa; 6 Department of Human Anatomy, Faculty of Medicine, Umm Al-Qura University, Makkah 24382, Saudi Arabia; agkushi@uqu.edu.sa; 7 Department of Internal Medicine, College of Medicine, Umm Al-Qura University, Makkah 24382, Saudi Arabia; aasarhan@uqu.edu.sa; 8 Department of Histology, Faculty of Medicine, Kafrelsheikh University, Kafr Elsheikh 33511, Egypt; mero.osman@med.kfs.edu.eg; 9 Department of Anatomy and Embryology, Faculty of Medicine, Kafrelsheikh University, Kafr Elsheikh 33511, Egypt; amira_sm201161@yahoo.com

In the original publication [1], there were mistakes in Figures 4 and 9 as published. In the legend for Figures 4 and 9, we corrected the legend for the “control group” in Figure 4A, and the “treated group DOX + CeNPs” in Figure 9D,I. We used the wrong image for the “control group” in Figure 4A, and for the “treated group DOX + CeNPs” in Figure 9D. The corrected Figure 4 and Figure 9 appear below. The authors state that the scientific conclusions are unaffected. This correction was approved by the Academic Editor. The original publication has also been updated.

## Figures and Tables

**Figure 4:**
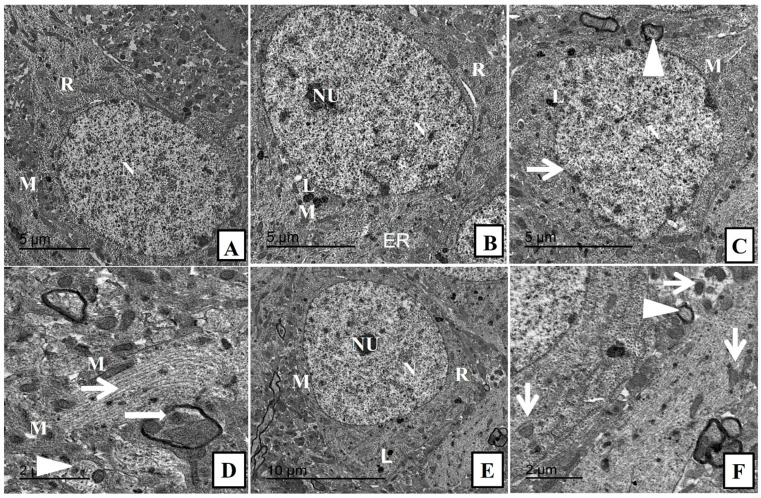
Transmission electron micrographs of hippocampus from different groups. (**A**) control group showing the normal structure of the neuron with normal nucleus (N), and cytoplasm containing mitochondria (M), and ribosome (R). (**B**) CeNPs group showing the normal structure of the neuronal cells with intact nuclear envelope, distinct nucleoli, normal cytoplasmic organelles (mitochondria (M), ribosomes (R), lysosomes (L), and endoplasmic reticulum (ER)). (**C**) DOX group showing enlarged nucleus with blebs of nuclear envelope (thin arrow), loss of nucleolus, few irregular shaped mitochondria (M) with minimal degenerating sheaths surrounding a degenerated axon (arrowhead). (**D**) DOX group showing completely loss of myelin sheath (arrowhead), other area showing intact axon with gradual loss of myelin (thick arrow) beside dilated microtubules (thin arrow) intercalated with few irregular shaped mitochondria (M). (**E**,**F**) Treated group Dox + CeNPs showing the normal architecture of nucleus (N) with prominent nucleoli (NU) and most cytoplasmic organelles (mitochondria (M), lysosome (L), ribosome (R) within the neurological cell. (**F**) Higher power showing with minimal mitochondrial membrane losses (thin arrows) either inside and around the neuronal cell with minimally affected sheath (arrowhead). Scale bar = 5 µm (**A**–**C**), 2 µm (**D**,**F**), 10 µm (**E**).

**Figure 9:**
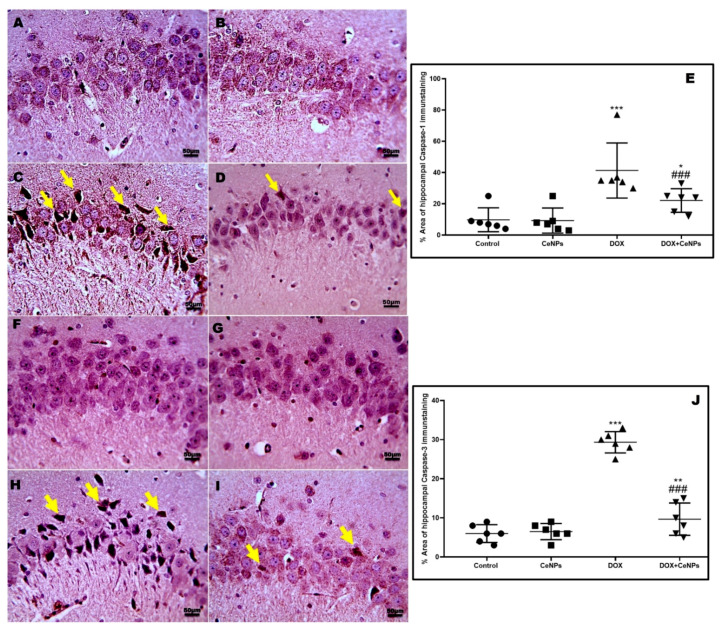
Effect of nanoceria on the hippocampal expression of caspase 1 and caspase 3 enzymes. (**A**,**B**,**F**,**G**) Hippocampal sections of control group and group which received CeNPs showing few numbers of positively brown stained neurons in pyramidal layer in CA3 region. (**C**,**H**) Hippocampal sections from DOX group showed markedly increased numbers of positively brown stained neurons (yellow arrows). (**D**,**I**) Hippocampal sections from treated group DOX + CeNPs showed decreased numbers of positively brown stained neurons (yellow arrows). (**E**,**J**). Histogram of % area of immunostaining hippocampal caspase 1 and caspase 3, respectively. Values are expressed as mean ± SD. The statistical investigation was carried out utilizing one-way ANOVA with Tukey’s post hoc analysis. *** *p* < 0.001, ** *p* < 0.01, and * *p* < 0.05 significant vs. control group ^###^ *p* < 0.001 significant vs. DOX group. Magnification ×400. Scale bar = 50 µm.
